# The Antiosteoporotic Activity of Central-Icaritin (CIT) on Bone Metabolism of Ovariectomized Rats

**DOI:** 10.3390/molecules191118690

**Published:** 2014-11-14

**Authors:** Jun Jiang, Jie Li, Xiaobin Jia

**Affiliations:** 1 Affiliated Hospital on Integration of Chinese and Western Medicine, Nanjing University of Chinese Medicine, Nanjing 210023, Jiangsu Province, China; E-Mail: xuyan9323@126.com; 2 Key Laboratory of New Drug Delivery System of Chinese Materia Medica, Jiangsu Provincial Academy of Chinese Medicine, 100# Shizi Road, Nanjing 210028, Jiangsu Province, China; E-Mail: lijiejstcm@163.com

**Keywords:** central-icaritin (CIT), antiosteoporotic activity, ovariectomized rats

## Abstract

Central-icaritin (CIT) is a flavonoid aglycone first discovered in our laboratory, which is an isomeric aglycone of icaritin (IT). We wanted to know whether CIT also had anti-osteoporosis activity. In this study, CIT was investigated in an ovariectomized rat (OVX) model. Fifty-six 6-month old female Sprague-Dawley rats were randomly assigned to sham operated group (Sham) and six OVX subgroups (*n* = 8 each). The OVX rats were then subdivided into six groups treated with vehicle (OVX), icaritin (IT, 40 mg/kg body weight/day), estradiol valerate (EV, 100 μg/kg body weight/day) or CIT (10, 20, and 40 mg/kg body weight/day) for 12 weeks, respectively. Then, the serum biochemical parameters, bone mineral density (BMD), bone biomechanical properties, bone microarchitecture, bone immunohistochemistry and related protein and gene expressions were evaluated. In OVX rats, the increases of body weight, HOP, AKP, and TRACP5b levels, and the decreases of uterus wet weight, femurs weight, BMD, serum OPG/RANKL and OCN were significantly inhibited by CIT treatment. Micro-CT analysis results showed that CIT apparently enhanced trabecular bone compared with the OVX group (*p* < 0.05). Total femur BMD and biomechanical strength of tibia were significantly improved (*p* < 0.05) after 12 weeks of CIT administration. In addition, the CIT administration also significantly enhanced the OPG expression, whereas reduced the RANKL expression in femurs according to RT-PCR, western blot assays and immunohistochemical evaluation. CIT had the antiosteoporotic activity, and its antiosteoporotic effects in OVX rats may be stronger than that of IT.

## 1. Introduction

Postmenopausal osteoporosis has been regarded as a heterogeneous disorder disease characterized by increased risks of bone fracture and bone loss deriving from an imbalance between osteoblast-mediated bone formation and osteoclast-mediated bone resorption [1]. A mounting number of investigations have demonstrated that decreased concentrations of circulating estrogen cause osteoporosis and induce bone loss. This fact has been confirmed by hormone replacement therapy (HRT) which is able to ameliorate the loss of estrogen at menopause and thus prevent postmenopausal osteoporosis [2]. However, the higher incidence of endometrial cancer, mammary cancer, and increased risk of coronary heart disease or cerebrovascular accident diseases caused by HRT exceed its benefits [3,4,5]. At present, bisphosphonates (BPs) and RANK Ligand antibodies are approved anti-resorptive therapies that reduce risk of fracture better than HRT, with no side effects related to estrogenicity [6]. In order to find new anti-osteoporosis active ingredients, many researchers have turned to the search for alternative or natural phytoestrogen-based therapies for osteoporosis.

Epimedium has been frequently prescribed for the treatment of osteoporosis in China for a long time [7]. Epimedium-derived flavonoids (EFs), including icariin and icaritin, display the capacity to exert beneficial effects on osteoporotic bone in postmenopausal women without side effects on reproductive tissues [8,9]. Moreover, the potency of *in vitro* estrogenic activity was in the order of icaritin > icariin [10]. Therefore, we speculated that the aglycones of Epimedium flavonoids might have better anti-osteoporosis effects than the corresponding glycosides.

**Figure 1:**
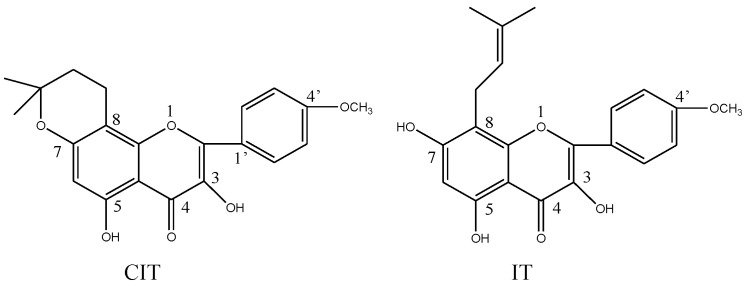
Chemical structures of CIT and IT.

In our initial research, we wanted to preparing IT, and do some related research. Unexpectedly, we found that the structure of our prepared aglycone (central-icaritin, CIT) was not consistent with existing reports after its structural identification. In this case, we decided to investigate the pharmacological effects of CIT, and compare its activity with IT. CIT, C_21_H_20_O_6_, 3,5-dihydroxy-2-(4-methoxyphenyl)-8,8-dimethyl-9,10-dihydro-8*H*-pyrano[2,3-f]chromen-4-one, an isomeric aglycone of icaritin, was first discovered and prepared in our laboratory (Figure 1). Since IT had anti-osteoporosis, activity, we were interested in determining if its isomer CIT also had anti-osteoporosis effects, hence the studies reported here.

## 2. Results and Discussion

### 2.1. Body Weight, Femur Weight and Uterus Wet Weight Changes in the OVX Rats

**Figure 2:**
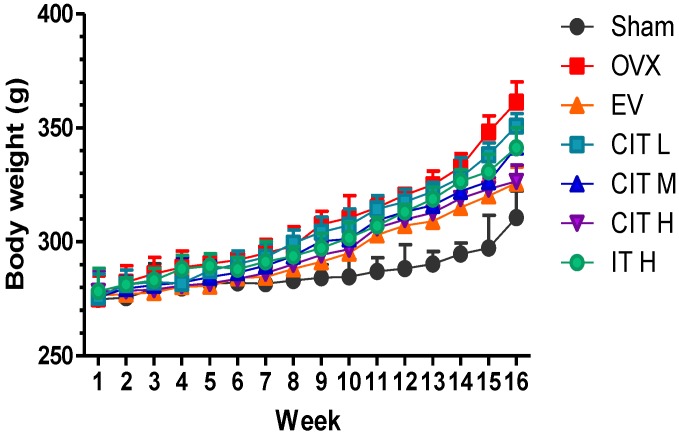
Body weight changes in the OVX model of osteoporosis. OVX rats received no treatment (OVX), EV (100 μg/kg/day), CIT (10, 20 and 40 mg/kg/day), and IT (40 mg/kg/day) for 12 weeks. Values were mean ± SD, error bars in the figure are presented as SD, *n* = 8. OVX compared with Sham *p* < 0.01, EV, CIT-H, CIT-M, IT compared with OVX *p* < 0.05, CIT-H compared with IT *p* < 0.05.

The average body weights of rats in each group throughout the experimental period are depicted in Figure 2. There was no significant difference in the mean body weight initially in each group. However, the rats in the EV, CIT-H and CIT-M groups weighed significantly less than the OVX rats (*p* < 0.05) after 12 weeks. The body weight of the CIT groups at all doses (CIT-H, CIT-M and CIT-L) increased less than the OVX group, and were decreased by 9.67% (*p* < 0.01), 5.54% (*p* < 0.05) or 3.05%, respectively, compared with the OVX group. The body weights of the rats in the EV and IT group were also lower than the OVX group by 9.97% and 5.54% (*p* < 0.05). The rats in thhe CIT-H groups weighed significantly less than the IT rats (*p* < 0.05).

As shown in Table 1, the femur weight and uterine wet weight differences between the sham and OVX rats were significant (*p* < 0.01). The femur weight and uterine wet weight of the CIT-H group was higher than the OVX group by 14.04% and 339.81%, and the differences were significant (*p* < 0.01). Similarly, the EV and IT treated groups demonstrated a significantly enhanced femur weight and uterine wet weight by 20.65%, 563.11% and 10.66%, 259.22% (*p* < 0.001) as compared with the OVX group. The femur weight and uterine wet weight of the CIT-H group were higher than in the IT group, and the differences were significant (*p* < 0.05).

### 2.2. Serum Biochemical Markers Changes in the OVX Rats

**Figure 3:**
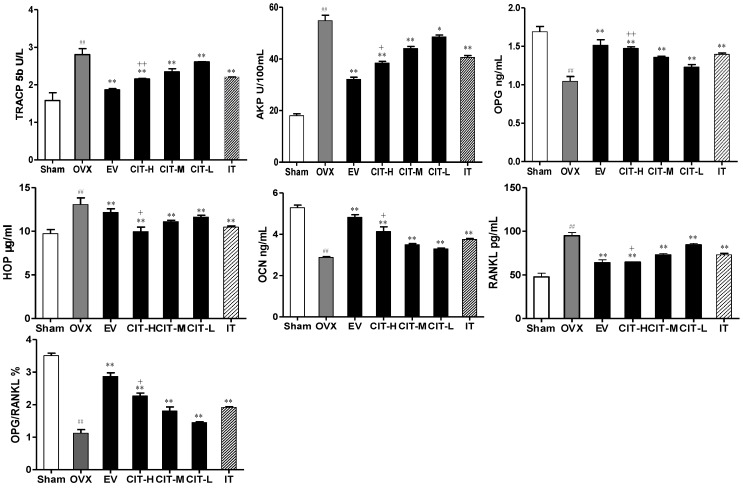
Serum parameters changes in the OVX model of osteoporosis. OVX rats received vehicle (OVX), EV (100 μg/kg/day), CIT (10, 20 and 40 mg/kg/day), and IT (40 mg/kg/day) for 12 weeks. Values are mean ± SD, error bars in the figures were presented as SD, *n* = 8. ^##^ *p* < 0.01 compared with sham, ^**^ *p* < 0.01 compared with OVX, ^+^ *p* < 0.05 compared with IT, ^++^ *p* < 0.01 compared with IT.

The effects of the CIT on serum biochemical parameters of osteoporosis in rats after 12 weeks are shown in Figure 3. OVX rats showed significantly increased (*p* < 0.01) levels of serum HOP, AKP, TRACP5b and RANKL by 34.33%, 199.78%, 77.22% and 99.29% compared with sham group. Treatment with CIT, EV, and IT significantly prevented the elevation of serum HOP, AKP, TRACP5b and RANKL levels compared with the OVX group (*p* < 0.01). Meanwhile, OVX rats showed a significant decrease (*p* < 0.01) in levels of serum OPG, and OCN by 38.09%, 45.27% compared with the sham group. Treatment with CIT, EV, and IT significantly increased the elevation of serum OPG, and OCN levels compared with the OVX group (*p* < 0.01).

**Table 1:** The comparation of femur weight and uterine wet weight.

Weight	Sham	OVX	EV	CIT-L	CIT-M	CIT-H	IT
Uterine (g)	0.783 ± 0.067	0.103 ± 0.015 ^##^	0.683 ± 0.031 ^**^	0.127 ± 0.015	0.233 ± 0.051 ^*^	0.453 ± 0.031 ^**+^	0.370 ± 0.02 ^**^
Femur (g)	0.971 ± 0.040	0.741 ± 0.025 ^##^	0.894 ± 0.016 ^**^	0.777 ± 0.006	0.815 ± 0.017 ^*^	0.845 ± 0.007 ^**^^+^	0.820 ± 0.01 ^**^

Notes: The data were expressed as mean ± SD; ^##^ *p* < 0.01 compared with sham; ^*^ *p* < 0.05 compared withOVX; ^**^ *p* < 0.01 compared with OVX; ^+^ *p* <0.05 compared with IT.

The serum HOP, AKP, TRACP5b and RANKL levels of the CIT-H group was lower than those of the IT group, and the difference was significant (*p* < 0.05). The serum OPG, and OCN levels of the CIT-H group was higher than that of the IT group, and the difference was significant (*p* < 0.05).

### 2.3. Bone Mineral Density (BMD) Changes in the OVX rats

As shown in Table 2, the right total femora BMD of OVX rats declined significantly to 357.63 ± 9.26 mg/cm^3^ compared with the 503.05 ± 16.52 mg/cm^3^ value of sham rats (*p* < 0.01), indicating that the OVX decreased the BMD by 28.84% after 12 weeks. In the same period of time, CIT at a dosage of 20 (CIT-M) or 40 mg/kg (CIT-H) notably increased the BMD by 5.88% (*p* < 0.05) or 9.49% (*p* < 0.01), respectively, compared with the OVX group. Similarly, EV and IT treated rats also increased total femora BMD by 20.39% and 5.87% compared with OVX rats. Furthermore, significant differences were observed among CIT groups. The BMD of the CIT-H (40 mg/kg/day) group was higher than that of the IT (40 mg/kg/day) group, and the difference was significant (*p* < 0.05).

### 2.4. Micro-CT Analysis in the OVX Rats

**Figure 4:**
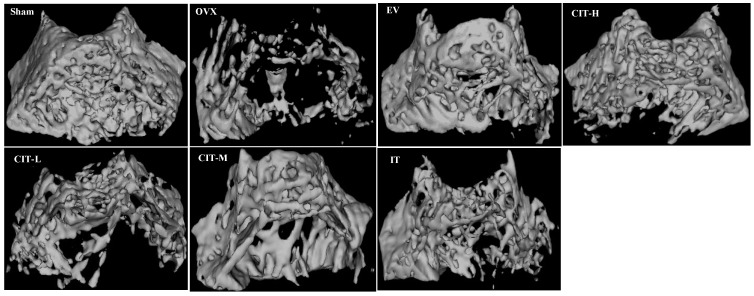
Representative Micro-CT images of trabecular bone microarchitecture in the distal femurs. The OVX rats presented notable reduction in the trabecular number, trabecular area compared with the Sham rats. CIT, EV and IT partially prevented OVX-induced trabecular bone loss and significantly improved trabecular bone mass and microarchitecture. CIT significantly improved trabecular bone microarchitecture compared with IT.

The quantitative results are expressed as BMC, TMC, TMD, VOB, Calib.Tb.Th.3D, Calib.Tb.Sp.3D, BV/TV, BS/BV, Tb.N, Tb.Sp and Tb.Th in Table 2. Analysis of the distal femur morphometric parameters indicated a deterioration of the microarchitecture in the OVX group compared with the sham group, which was evidenced by significant decreases of trabecular BMC, TMC, TMD, VOB, BV/TV, Tb.Th, Tb.N and Calib.Tb.Th.3D (*p* < 0.01) and significant increases of Tb.Sp, BS/BV and Calib.Tb.Sp.3D (*p* < 0.01). Treatment with CIT, EV, and IT reversed the abovementioned parameters and all were significant as compared with OVX group (*p* < 0.05) except Calib.Tb.Th.3D in the CIT-L group. Meanwhile, the preventive effects of CIT on trabecular bone mass and microarchitecture deterioration were enhanced with its increasing doses, and which were further proved by the 3D Micro-CT images (Figure 4). The OVX group presented a conspicuous reduction in the trabecular number and trabecular area when compared with the sham group. CIT partially prevented OVX-induced bone loss and significantly improved the trabecular bone mass and microarchitecture after 12 weeks treatment.

**Table 2:** BMD and Micro-CT analysis of BMC, TMC, TMD, VOB, BS/BV, BV/TV, Tb.Th, Tb.Sp, Tb.N, Calib.Tb.Th.3D and Calib.Tb.Sp.3D.

Parameters	Sham	OVX	EV	CIT-L	CIT-M	CIT-H	IT
BMD (mg/cm^3^)	503.05 ± 16.52	357.63 ± 9.26 ^##^	431.85 ± 14.50 ^*^	366.41 ± 9.90 ^*^	379.42 ± 8.02 ^*^	392.66 ± 4.58 ^**+^	379.12 ± 2.92 ^*^
BMC (mg)	263.84 ± 5.25	147.03 ± 6.47 ^##^	246.15 ± 10.02 ^**^	182.172 ± 4.39 ^**^	204.09 ± 7.36 ^**^	222.08 ± 3.39 ^**+^	203.53 ± 5.71 ^**^
TMC (mg)	245.16 ± 4.57	131.83 ± 3.33 ^##^	227.08 ± 3.17 ^**^	163.56 ± 6.53 ^**^	180.23 ± 8.08 ^**^	201.52 ± 3.16 ^**+^	193.90 ± 3.01 ^**^
TMD (mg/cm^3^)	587.37 ± 2.92	488.14 ± 4.62 ^##^	579.00 ± 4.77 ^**^	509.87 ± 0.10 ^**^	515.86 ± 5.49 ^**^	554.44 ± 7.37 ^**++^	591.33 ± 6.06 ^**^
VOB (mm^3^)	448.08 ± 16.78	241.25 ±17.07 ^##^	415.75 ±12.94 ^**^	312.46 ±17.31 ^**^	358.19 ±10.16 ^**^	382.53 ± 8.98 ^**+^	357.97 ± 11.73 ^**^
BS/BV (1/mm)	2.71 ± 0.09	5.44 ± 0.11 ^##^	3.41 ± 0.05 ^**^	4.55 ± 0.09 ^**^	4.06 ± 0.08 ^**^	3.77 ± 0.07 ^**+^	3.86 ± 0.06 ^**^
BV/TV (%)	59.95 ± 4.26	28.56 ± 1.02 ^##^	54.31 ± 4.19 ^**^	39.20 ± 1.22 ^**^	44.36 ± 1.16 ^**^	46.11 ± 0.84 ^**+^	44.42 ± 0.44 ^**^
Tb.Th (mm)	0.71 ± 0.02	0.33 ± 0.02 ^##^	0.64 ± 0.03 ^**^	0.36 ± 0.01 ^*^	0.44 ± 0.01 ^**^	0.47 ± 0.02 ^**+^	0.43 ± 0.01 ^**^
Tp.Sp (mm)	0.24 ± 0.01	0.69 ± 0.03 ^##^	0.32 ± 0.01 ^**^	0.61 ± 0.01 ^*^	0.45 ± 0.01 ^**^	0.41 ± 0.02 ^**+^	0.45 ± 0.02 ^**^
Tb.N (1/mm)	6.11 ± 0.91	1.68 ± 0.06 ^##^	4.13 ± 0.32 ^**^	2.01 ± 0.13 ^*^	2.74 ± 0.12 ^**^	3.65 ± 0.17 ^**+^	3.20 ± 0.12 ^**^
Calib.Tb.Th (mm)	0.76 ± 0.04	0.44 ± 0.04 ^##^	0.67 ± 0.01 ^**^	0.50 ± 0.01	0.57 ± 0.01 ^*^	0.61 ± 0.02 ^*+^	0.58 ± 0.01 ^**^
Calib.Tb.Sp (mm)	0.22 ± 0.01	0.58 ± 0.01 ^##^	0.30 ±0.01^**^	0.53 ± 0.01 ^**^	0.42 ± 0.01 ^**^	0.35 ± 0.02 ^**++^	0.41 ± 0.01 ^**^

Notes: The data were expressed as mean ± SD; ^##^ *p* < 0.01 compared with Sham; ^*^ *p <* 0.05; ^**^ *p* < 0.01; compared with OVX; ^+^ *p* < 0.05; ^++^ *p* < 0.01 compared with IT.

**Table 3:** Bone biomechanical parameters by three-point bending test.

Parameters	Sham	OVX	EV	CIT-L	CIT-M	CIT-H	IT
Ultimate load (N)	94.66 ± 3.99	63.11 ± 2.69 ^##^	88.76 ± 1.72 ^**^	68.38 ± 1.73 ^*^	76.10 ± 1.57 ^*^	81.47 ± 1.94 ^*+^	75.56 ± 1.26 ^**^
Stiffness (N/mm)	247.27 ± 17.20	146.29 ± 12.33 ^##^	224.02 ± 6.38 ^**^	164.37 ± 10.87	177.48 ± 8.53 ^*^	196.25 ± 7.03 ^**+^	180.69 ± 5.53 ^**^
Energy to failure (mJ)	23.37 ± 2.02	11.51 ± 1.16 ^##^	21.07 ± 0.62 ^**^	13.99 ± 1.00 ^*^	16.94 ± 0.98 ^**^	18.92 ± 0.47 ^**+^	16.84 ± 1.10 ^**^

Notes: The data were expressed as mean ± SD; ^##^ *p* < 0.01 compared with Sham; ^*^ *p* < 0.05, ^**^ *p* < 0.01 compared with OVX; ^+^ *p* < 0.05 compared with IT.

### 2.5. Three-Point Bending Test in the OVX Rats

The effects of CIT on biomechanical parameters of osteoporosis in rats after 12 weeks using a three-point bending experiment are shown in Table 3. After twelve weeks of estrogen deficiency, the biomechanical parameters of tibia, such as ultimate load, stiffness, and energy absorption, decreased significantly in the OVX group as compared with the sham group (*p* < 0.01). A significant increase in biomechanical strength was observed in the CIT, IT and EV treatment groups as compared with the OVX group (*p* < 0.05), evidenced by increased levels of ultimate load, stiffness, and energy absorption. Moreover, the CIT-H treated group demonstrated a significant enhancment of the ultimate load, stiffness, and energy absorption levels (*p* < 0.05) as compared with the IT group.

**Figure 5:**
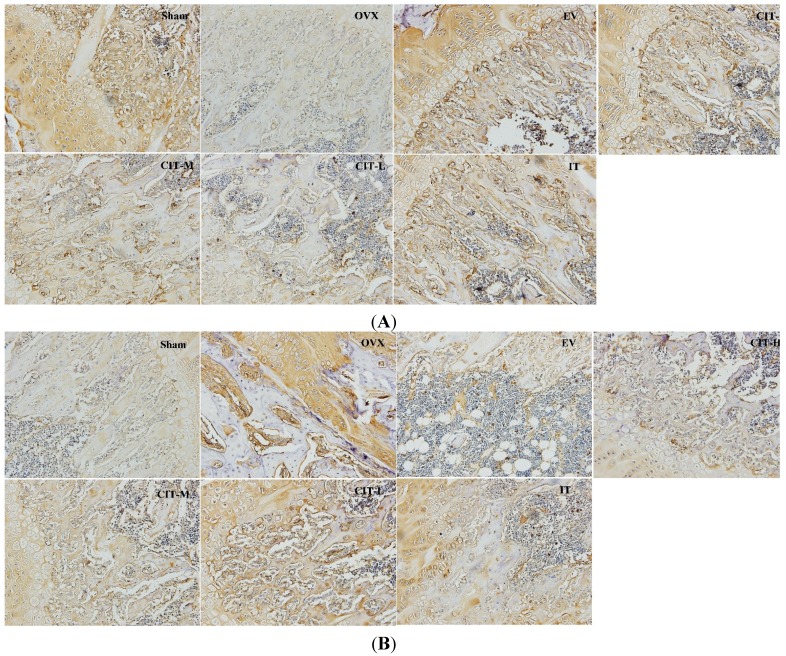
Immunohistochemical staining of osteoporotic rats’ femurs. (**A**) OPG staining, OPG protein stain is brown. The OVX rats presented notable reduction in OPG expression compared with the sham rats. CIT, EV and IT significantly improved OPG expression compared with OVX rats; (**B**) RANKL staining, RANKL protein stain is brown. The OVX rats showed a significant increase in RANKL expression compared with the sham rats. CIT, EV and IT significantly inhibited RANKL expression compared with OVX rats.

### 2.6. Immunohistochemical Analysis in the OVX Rats

The OPG and RANKL expressions appeared as yellow-brown staining in the cytoplasm (Figure 5). Optical microscopy images were used for OPG or RANKL staining. A decrease in OPG labeling was already observed in the OVX group compared with the sham group, whereas the OPG expression of the CIT-treated groups were higher than that of the OVX group after 12 weeks administration, similar to the IT and EV-treated groups. CIT-M group and CIT-H group significantly increased the OPG expression compared with OVX group. A significant increase in RANKL labeling was observed in the OVX group compared with the sham group, whereas the RANKL expression of the CIT-treated groups were lower than that of the OVX group after 12 weeks administration, similar to the IT and EV-treated groups. CIT-M and CIT-H groups showed significantly decreased RANKL expression compared with the OVX group.

**Figure 6:**
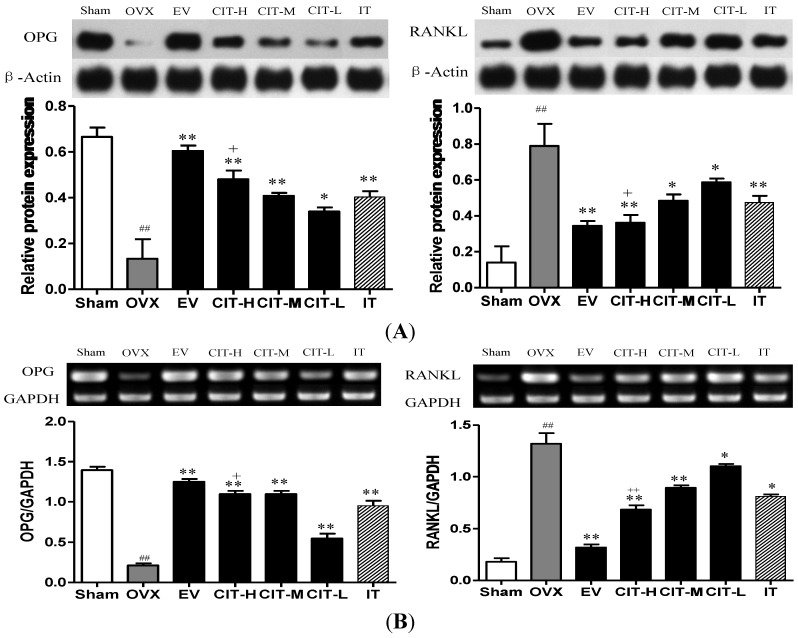
Related proteins and genes expressions in osteoporotic rats femur. (**A**) OPG and RANKL protein expressions in femur of OVX rats. Effects of 12-week treatment with EV, CIT and IT on protein expressions of osteoprotegerin (OPG) and receptor activator of nuclear factor kappa-B ligand (RANKL); (**B**) OPG and RANKL mRNA expressions in femur of OVX rats treated with EV, CIT and IT for 12 weeks. Total RNA was isolated and RT-PCR was performed to determine OPG and RANKL mRNA expressions. Values are expressed as means ± SD (*n* = 8); ^##^ *p* < 0.01 compared with sham, ^*^ *p* < 0.05, ^**^ *p* < 0.01 compared with OVX, ^+^ *p* < 0.05, ^++^ *p* < 0.01 compared with IT group.

### 2.7. Expressions of OPG and RANKL in the OVX Rats

Protein and mRNA expressions of OPG were decreased significantly in tibia tissue from OVX rats compared with the sham group (*p* < 0.01). The CIT, EV and IT treatment increased the expressions of OPG protein and mRNA in tibia of OVX rats compared with the OVX group (*p* < 0.05). Ovariectomy caused a significant increase of protein and mRNA expressions of RANKL in tibia compared with the sham group (*p* < 0.01). Protein or mRNA expressions of RANKL in tibia from OVX rats decreased significantly after treatment of CIT, IT, and EV (*p* < 0.05). Furthermore, the CIT-H treatment group displayed significantly decreased protein and mRNA expressions of RANKL, and significantly increased protein and mRNA expressions of OPG compared with the IT group (*p* < 0.05) (Figure 6).

### 2.8. Discussion

In this paper, we explored the effects of CIT on OVX-induced osteoporosis in rats [11,12]. Our study clearly demonstrates the beneficial effects of CIT on the prevention of bone loss which is associated with ovariectomy. It is well known that estrogen deficiencies are important risk factors in the pathogenesis of osteoporosis. Ovariectomy results in a dramatic decrease in uterine wet weight, femur weight, BMD, biomechanical strength, and bone quality, and these changes are due to estrogen deficiency [13]. Our data showed that OVX decreased the weight of the uterus and femurs, and increased the body weight when compared with the sham group (*p* < 0.05), clearly showing the effectiveness of ovariectomy (Table 1). CIT treatments significantly increased the uterine weight and femur weight, and serum OPG and OCN levels, as well as decreased body weight and the serum HOP, AKP, TRACP5b and RANKL levels in OVX rats. In line with this finding, CIT administration also significantly enhanced the expression of OPG and decreased the expression of RANKL in the femur according to immunohistochemical evaluation. Moreover, the CIT treatment significantly enhanced total femoral BMD, biomechanical strength, and bone quality for 12 weeks. These results suggested that CIT had a similar effect as estrogen on uterine weight, femur weight, serum HOP, AKP, TRACP5b, RANKL, OPG and OCN levels. It demonstrated that CIT possessed antiosteoporotic activity in osteoporotic rats induced by estrogen deficiency. Therefore, CIT could be a potential therapeutic anti-osteoporosis drugr.

BMD has been described as an important and primary contributor to bone quality [14]. BMD is markedly decreased due to an increase in bone turnover in the OVX rats compared with the sham group. In our study, CIT significantly improved the BMD of the OVX group, which indicated that CIT could prevent the progress of bone loss induced by estrogen deficiency (Table 2). Moreover, CIT treatment increases the BMD in OVX rats in a dose-dependent manner. Furthermore, the three point bending test results supported the BMD results, which were evidenced by improving bone mechanical properties such as ultimate load (N), stiffness (N/mm) and energy absorption (mJ) (Table 3). In addition, the trabecular bone microarchitecture quantified by Micro-CT might improve the estimation of bone strength [15].

Serum HOP, AKP, TRACP5b, RANK, OPG and OCN are known to be important biochemical markers and indicators of osteogenic properties. The levels of HOP, AKP, TRACP5b and RANKL were increased while OPG and OCN levels were decreased in osteoporosis due to increased bone turnover [16]. These factors also supported the observations of other investigators that these changes were due to ovarian hormone deficiency and were prevented by estrogen administration [17]. Our study demonstrated that both EV and CIT inhibited OVX-induced enhancement of serum HOP, AKP, TRACP5b and RANKL levels, and promoted OVX-induced reduction of serum OPG and OCN levels. In this study, treatment with EV and CIT almost reversed the changes of serum HOP, AKP, TRACP5b, RANKL, OPG and OCN levels in OVX rats.

The coordination between osteoblast and osteoclast is a critical factor in the maintenance of skeletal integrity. The modulation of osteoclastogenesis by immature cells of the osteoblastic lineage is mediated by RANKL and osteoprotegerin (OPG) [16]. OPG is a decoy receptor that inhibits RANKL activation of osteoclastogenesis, thereby decreasing bone resorption. The ratio of OPG/RANKL expression is believed to be a key parameter of osteoclastogenic activity [17]. We found that CIT increased OPG protein and mRNA expressions, and reduced RANKL protein and mRNA expressions, suggesting that CIT inhibits osteoclast differentiation by increasing the OPG/RANKL ratio.

CIT and IT are isomeric flavonoid aglycones, and the chemical structure of CIT possesses a pyran ring, which is formed by the hydroxyl group at C-7 and isopentenyl group on C-8 compared with IT. Therefore, the isopentenyl group in IT has disappeared in the structure of CIT, while CIT loses one phenolic hydroxyl group compared with IT. IT exerts an anti-resorptive effect on osteoporotic bone [8]. Our results indicated that CIT had a stronger effect than IT on preventing estrogen deficiency osteoporosis, which were evidenced by preventing bone loss or preserving bone mass, improving BMD and repairing the femur bone microarchitecture.

The strongest potency of *in vitro* estrogenic activity was displayed by IT [10]. Meanwhile, *in vitro* experiments found that icaritin (IT) markedly enhanced the proliferation of MCF-7 cells, but not icariin [18]. The result inferred that only the non-conjugated IT form exerts estrogen-like activities. Our previous study found that *in vivo* icariin and epimedin B (main active ingredients in Epimedium) were mainly metabolized into IT in rats plasma, possibly by metabolizing enzymes or intestinal bacteria [19,20,21,22]. Therefore, we hypothesized that IT exerted its anti-osteoporotic effects in the body in the parent form rather than its metabolites or conjugates. However, IT could be easily absorbed into the body, and then rapidly conversed to its conjugated or metabolites, and finally removed from the body mainly via biliary excretion and the feces [23].

Therefore, we speculate that the number of phenolic hydroxyls in the structure of flavonoids can affect drug absorption, distribution, metabolism and speed of excretion in the body, and ultimately lead to the differences in pharmacological effects. In the structure of IT, there are two phenolic hydroxyls on C-3 and C-7, respectively. However, there is only one phenolic hydroxyl on C-3 in the structure of CIT. Our previous study clearly shown that IT could be rapidly conversed to its conjugates after being absorbed into the blood, which made the IT prototype lose its anti-osteoporosis activity and be rapidly excreted. In addition, the *in vivo* process of CIT involved enterohepatic circulation, which further extends its anti-osteoporosis efficacy role. Finally, CIT probably had better therapeutic effects than IT during the long-term continuous administration, which was mainly attributed to the smaller number of hydroxyl groups in the structure of CIT compared with IT [24].

## 3. Experimental Section

### 3.1. Animals and Reagents

Animal experiments were carried out according to the Guide for the Humane Use and Care of Laboratory Animals and were approved by the Animal Ethics Committee of Jiangsu Provincial Academy of Chinese Medicine. Fifty-six 6 months old female Sprague-Dawley rats with the body weight of 280 ± 20 g, were allowed to acclimatize for 14 days before the start of the experiment. Every four rats were kept in one cage with a standard laboratory rodent diet (calcium content 0.5%) and tap water under climate-controlled conditions (25 °C, 55% humidity, and 12 h of light alternating with 12 h of darkness). After 14 days of acclimatization, the rats were anesthetized with intraperitoneal (i.p.) injection of 300 mg/kg chloral hydrate (Sinopharm^®^, Beijing, China) and underwent either bilateral laparotomy (sham, *n* = 8) or bilateral OVX (OVX, *n* = 48). The surgical procedure was performed under aseptic conditions. Rats were left untreated for 4 weeks to allow rats to recover and develop osteopenia [25]. After 4 weeks, the OVX rats were randomly divided into six groups: ovariectomized treated with vehicle (OVX, *n* = 8), OVX treated with estradiol valerate (EV, *n* = 8, 100 μg/kg body weight/day), OVX treated with IT (IT, *n* = 8, 40 mg/kg body weight/day), and OVX treated with low dosage CIT (CIT-L, *n* = 8, 10 mg/kg body weight/day), with medium dosage CIT (CIT-M, *n* = 8, 20 mg/kg body weight/day), or with high dosage CIT (CIT-H, *n* = 8, 40 mg/kg body weight/day). EV, IT and CIT were suspended in 0.5% sodium carboxymethyl cellulose and administrated orally, which started from 5th week (29th day) after OVX to 16th weeks (112th day). Body weight was measured weekly, and the EV, IT and CIT dose were adjusted according to their body weight. After 12 weeks of intervention and on the last day of treatment, all rats were anesthetized with i.p. injection of 300 mg/kg chloral hydrate, and blood was collected from the carotid artery and allowed to clot, followed by centrifugation at 3000*×* g for 10 min. Serum was harvested and stored at −20 °C until biochemical assays. And then, the femur weight and uterus wet weight were recorded after removing their adherent connective tissues. Accordance with the order, right femur were prepared to detect BMD, scanned by microcomputed tomography, and then western blot assays. Left femur were used for quantifying gene expression by real-time PCR. Right tibia were estimated by three-point bending test. Left tibia were examined by immunohistochemistry. 

### 3.2. Serum Parameters

Serum samples were subjected for the measurement of serum hydroxyproline (HOP, A030-2) and alkaline phosphatase (AKP, A059-1) using commercial assay kits (Nanjing Jiancheng Bioengineering Institute, Nanjing, China) for the *in vitro* determination. The levels of osteoprotegerin (OPG, R046-2), Osteocalcin (OCN, R035), receptor activator of nuclear factor kB ligand (RANKL, R085-2), and tartrate-resistant acid phosphatase 5b (TRACP5b, R052-2) were determined with a sandwich enzyme-linked immunosorbent assay (ELISA) assay kit (R&D Systems Inc., Minneapolis, MN, USA). All the assays were performed according to manufacturer’s instructions. 

### 3.3. Measurement of Bone Mineral Density (BMD)

The BMD of the right total femur was measured by using dual energy X-ray Absorption Spectrometry (DEXA, Hologic Inc., Boston, MA, USA) equipped with software (Edition 13.1.2) using the small animal scan mode. The measurements obtained were expressed as milligram of mineral contents per cm^3^ of surface area.

### 3.4. Micro-CT Analysis

The right distal femora from each group were scanned by a Micro-CT system (eXplore Locus, GE Healthcare, Beijing, China) with Version MicroView ABA analysis software version 2.2 for scanning images reconstruction. The scanning system was set to 80 W, with an isotropic voxel size of 22 μm. Bone morphometric parameters including bone volume over total volume (BV/TV), trabecular number (Tb.N), trabecular separation (Tb.Sp), trabecular thickness (Tb.Th), bone mineral content (BMC), tissue mineral content (TMC), tissue mineral density (TMD), calibration of trabecular separation 3D (Calib.Tb.Sp.3D), calibration of trabecular thickness 3D (Calib.Tb.Th.3D), and volume of bone (VOB) were obtained by analyzing the volume of interest (VOI).

### 3.5. Biomechanical Evaluation

The freshly right tibias were isolated and assessed for the three-point bending test [26] using CSS-4420 material testing machine (Changchun Research Institute for Testing Machines Co. Ltd., Changchun, China). The force and displacement data were automatically recorded into a computer which was interfaced to the material testing machine and the load-deformation curve was plotted simultaneously. The results of measurements of ultimate load (Newtons, N), extrinsic stiffness (Newtons per millimeter, N/mm) and energy to yield point (mJ) were calculated with the load-deformation curve.

### 3.6. Immunohistochemical Staining

The left tibia from each group were fixed in 10% formalin for 2 days at room temperature, and then transferred into 10% ethylenediamine tetraacetic acid (EDTA) solution (pH 7.2–7.4), which was replaced once every three days for decalcification. One month later, the immunohistochemical evaluation was applied to analyze the OPG and RANKL expressions. For immunohistochemical labeling, cryosections were warmed to 65 °C for 5 min and then rehydrated through xylene and a series of graded ethanol solutions. Endogenous peroxidase was blocked by incubating sections with 0.3% H_2_O_2_ in PBS for 15 min. Sections were then washed in PBS for three times. The blockade was performed with 10% rabbit serum in PBS for 1 h at room temperature. Primary antibody anti-sclerostin (Goat IgG anti-mouse SOST Affinity Purified Ab, R&D Systems, diluted 1:200 with 2% rabbit serum in PBS) was incubated with the sections overnight at 4 °C. Secondary antibody (biotinylated rabbit anti-goat IgG diluted 1:200 with 2% rabbit serum in PBS) was then incubated with the sections for 1 h at room temperature. The slides were developed with VECTASTAIN Elite ABC reagents (Vector Laboratories, Burlingame, CA, USA) according to the manufacturer’s protocols.

### 3.7. Real-Time PCR

Total RNA was extracted from left femurs using a tissue homogenizer (PowerGen Model 1000, Fisher Scientific, Waltham, MA, USA) and TRI-reagent. It was then converted to cDNA by reverse transcriptase (High Capacity cDNA Reverse Transcription Kit, Applied Biosystems) using random hexamer. Real-time PCR amplification was performed with multiple samples using gene-specific PCR primers and gene-specific Taqman probes (Applied Biosystems). The PCR mixture (including Taqman primer) was run in an ABI PRISM 7500 Sequence Detection System (Applied Biosystems). Reagents used for the PCR reaction were purchased from Applied Biosystems. Each sample was run in duplicate in a volume of 20 μL using temperature cycling according to the manufacturer's protocols. The relative quantification of OPG and RANKL gene expressions were normalized to the expression of a housekeeping gene, glyceraldehyde 3-phosphate dehydrogenase (GAPDH). Data were expressed as fold change in mRNA level compared with a control group.

### 3.8. Western Blot Assays

Right femurs were homogenized in a cocktail of protease inhibitors (RIPA and HALT, Thermo Scientific) using a tissue homogenizer (PowerGen Model 1000, Fisher Scientific). The resultant mixture was centrifuged (13,000 g) for 10 min to pellet any bone fragments. Supernatants were collected and assayed for protein (Pierce BCA assay, Thermo Scientific). Equal amounts of protein (40 µg) were then diluted in sample buffer (62.5 mM Tris-HCl, pH 6.8, 10% (v/v) glycerol, 2% SDS, 0.1% bromophenol blue), and run in 10% SDS-PAGE gels. Samples were transferred onto nitrocellulose membranes by electroblotting (BioRad, Beijing, China). Filters were blocked for 2 h in a blocking buffer containing 5% powdered milk and TBS + 0.1% Tween. Filters were then incubated with a primary antibody OPG (1:1000) or RANKL (1:1000) at 4 °C overnight. After washing, membranes were incubated for 1 h with appropriate secondary antibodies (Rabbit anti-Goat, or Goat-anti-Rabbit, R&D Systems). The bands were developed by enhanced chemiluminescence using Chemiluminescence LumiGLO (Cell Signaling Technology, Waltham, MA, USA). The detection process was carried out by autoradiography. Quantification was achieved by digitalizing the blots using an imaging station and then measuring band density.

### 3.9. Statistical Analysis

The data were analyzed by one way analysis of variance (ANOVA). A Student Neuman-Keuls (SNK) post-hoc test will be conducted on the pooled data to determine the differences between the groups at a significance level of *p* < 0.05.

## 4. Conclusions

In conclusion, CIT treatment showed a remarkable antiosteoporotic activity on adult OVX rats. Therefore, CIT, as a natural derived flavonoid aglycone compound, could be developed into an effective agent for the prevention or treatment of osteoporosis. In order to develop CIT into a new drug for the treatment of osteoporosis, our further studies will be focused on the pharmacokinetics of CIT, including its bioavailability, absorption, distribution, metabolism, excretion and formulation design.
